# Differential expression of genes in the subgranular zone and granular cell layer of the hippocampus after running

**DOI:** 10.20463/jenb.2018.0025

**Published:** 2018-12-31

**Authors:** Hyo-youl Moon

**Affiliations:** 1 Department of Physical Education, Seoul National University, Seoul Republic of Korea; 2 Institute of Sport Science, Seoul National University, Seoul Republic of Korea

**Keywords:** Exercise, Tollip, subgranular zone, granular cell layer, hippocampus

## Abstract

**[Purpose]:**

Despite numerous studies, the mechanisms underlying the effects of exercise on brain function are not yet fully understood. Adult hippocampal neurogenesis is one of the most well-known effects of exercise on the brain, but its physiological roles during exercise are still ambiguous, mostly due to the difference in the structure and composition of each part of the hippocampus.

**[Methods]:**

In this study, we analyzed exercise-induced changes in gene expression in the subgranular zone (SGZ) and granular cell layer (GCL) of the hippocampus.

**[Results]:**

Surprisingly, only about 10% of changes were common to both areas. *Tollip* expression, which is altered in the SGZ and in Engrailed-2 mutant mice following exercise, did not change in the GCL. Tollip levels were not changed in the whole hippocampus after two weeks of treadmill exercise, but immunofluorescence analysis showed that Tollip and Ki-67 co-localize in the hippocampal dentate gyrus . Through siRNA knockdown experiments, we found that levels of DCX and cellular survival rates were decreased in Tollip-deficient Neuro2A cells.

**[Conclusion]:**

Taken together, these results suggest a role for Tollip in mediating the beneficial effects of exercise, probably affecting cellular health in the SGZ of the hippocampus.

## INTRODUCTION

Participating in physical activities has diverse health benefits, including the prevention of metabolic and neurodegenerative diseases^1-3^ . Recent studies have suggested that exercise reduces the symptoms and risk of Parkinson’s disease^4-6^, Alzheimer’s disease^7,8^ and even some genetic disorders^9-11^. The common feature in these diseases is impaired neurogenesis^12-14^. The effects of physical activity on neurogenesis have been intensively investigated^15,16^. Running increases the proliferation of adult neuronal progenitor cells^17^ and neuronal differentiation^18^ which affects mood control, learning, and memory^16,19^. Furthermore, both voluntary^18^ and involuntary exercise, such as treadmill running, may mediate neurogenesis by increasing neurotrophic factors^20,21^. However, the underlying molecular mechanism linking exercise and a reduction in neurodegenerative diseases remains elusive. One of the reasons for this difficulty is that different regions of the same tissue have a different genetic structure, and they participate in varied functions^22,23^.

Autism Spectrum Disorder (ASD) is not only characterized by difficulties with social interactions, communication, and limited and repetitive behavior, but also by a specific profile of temporary memory loss, including explicit search of previously experienced events^24^. ASDs are highly heterogeneous due to their genetic traits, but share some common features such as dysregulation of genes involved in neurogenesis^12^. Accumulated research suggests that physical activity has positive effects on academic learning and reduces stress in ASD^25,26^. Neurogenesis in the adult hippocampus contributes to the memory recall process in humans and other mammals^27,28^. 

The subgranular zone (SGZ) of the hippocampus contains adult neuronal progenitor cells that are involved in various developmental stages. It is located between the granule cell layer (GCL) and the hilus of the dentate gyrus (DG)^29^. Despite the different characteristics of the GCL and the SGZ^23^, technical limitations make it difficult to sensitively detect their separate physiological and biochemical changes during exercise. In this study, we compared gene array data from the GEO datasets, comparing the SGZ and GCL of the hippocampal dentate gyrus from mice subjected to 30 days of running^23^. Then we further analyzed the differentially expressed genes from the SGZ after exercise and genes changed in transcription factor engrailed-2 loss-of-function mice^30^, a model of autism spectrum disorder.

## METHODS

### Datasets

Autism and exercise were separately used as search terms in the GEO series (https://www.ncbi.nlm.nih.gov/geo/browse/?view=series). After filtering out the datasets which were not obtained from the hippocampus, the dataset accession number GDS6016 for Autistic disorders and GDS5005 for voluntary exercise were used to compare expression profiles. To identify genes that display marked differences in expression level, we used the web-based module, “compare 2 sets of samples”, in the NCBI dataset browser. Both analyses used two-tailed t-tests with a significance level below 0.01. For GDS5005 analysis, mouse SGZ (GSM977862, GSM977864, and GSM977866) and GCL (GSM977863, GSM977865, and GSM977867) hippocampi samples were selected as controls and SGZ (GSM977874, GSM977876, and GSM977878) and GCL (GSM977875, GSM977877, and GSM977879) samples from mice subjected to 30 days of running were used as the exercise group. For GDS6016 analysis, hippocampi samples from engrailed-2 (En2) wildtype mice (GSM1249165, GSM1249166, and GSM1249167) were used as controls and En2 mutant mouse samples (GSM1249168, GSM1249169, and GSM1249170) were used to represent ASD. Both datasets used different platforms and reference series: GPL7202 and GSE51612 in GDS6016, and GPL81 and GSE39697 in GDS5005.

### Animals

One-month-old C57Bl/6 male (*n*=5) and female (*n*=5) mice were purchased from Central Lab Animals Inc. Mice were individually housed in standard conditions with food and water ad libitum. A week later, mice were divided into two groups (sedentary and exercise). Exercised animals were acclaimed on a treadmill for 3 days, and ran on a treadmill for 14 days (at a speed of 14 m/min for 1 hour per day during the first week and 16 m/min for 1 hour per day at a 6 degree incline during the second week). At the end of the scheduled experiments, the mice were sacrificed after deep anesthesia with isofluorane (Henry Schein Animal Health, OH) with O2 and their brains were dissected. Hippocampi were collected and immediately frozen and stored at -80ºC. Animals were maintained in accordance with the National Institutes of Health guidelines. The experimental procedures were approved by the animal ethical review board of Seoul National University (IACUC-171229-2-2).

### Neuroblastoma (Neuro2A) cell culture

The mouse neuroblastoma cell line, Neuro2A, was purchased from the American Type Culture Collection (ATCC, Manassas, VA) and cultured in Dulbecco’s modified Eagles medium (DMEM; GIBCO) supplemented with 10% (v/v) fetal bovine serum (FBS; GIBCO) at 37°C in a humidified incubator (ThermoFisher) containing 5% CO_2_. Mouse *Tollip* siRNA (catalog no. sc-63333), and control scramble siRNA (catalog no. sc-37007) were purchased from Santa Cruz Biotechnology. A total of 2 × 10^4^ cells/well of Neuro2A were plated for 24 h, and siRNA transfection was conducted using Lipofectamine 2000 (Invitrogen) according to the manufacturer’s instructions.

### Cell senescence assay

Following the treatment period, the cells were washed, fixed, and stained for β-galactosidase (β-gal, a common marker for cell senescence) using X-gal staining solution (Cell Signaling Technology, Danvers, MA), then incubated overnight at 37°C (as per manufacturer's instructions). The cells were then examined under a microscope at both 10× and 20× magnification (TE200; Nikon Instruments) for the development of the X-gal stain (blue). Images were captured (MetaMorph, version 5.0) with a camera at 10× magnification to analyze the percentage of stained versus non-stained cells. An average of 304 ± 45 cells were counted per well.

### PCR array analysis

Total RNA was extracted from hippocampi using a total RNA extraction kit (Ribozol, Amresco) following manufacturer’s instructions. 5 μg of RNA in 10 μl was used for cDNA synthesis. First-strand cDNA was synthesized by RT using oligo (dT) primers and SuperScript II reverse transcriptase (Invitrogen). For the PCR array, total RNA (100 ng) was amplified using SYBR Green Master Mix (ThermoFisher) and analyzed using a CFX96 qPCR instrument (Bio-rad).

### Western blot analysis of brain tissue

Brain tissue samples were lysed with RIPA buffer (Millipore, CA) including Protease/Phosphatase Inhibitor Cocktail (Cell Signaling Technology, Danvers, MA) and sonicated with 1 second bursts for 15 seconds in ice. 300 µL of lysis buffer was added per ~5 mg of tissue. The lysates were centrifuged at 14,000 rpm for 15 min at 4°C. For western blot analysis, supernatant fractions containing equal amounts of protein were subjected to SDS-PAGE and transferred to nitrocellulose membranes (Millipore, MA). The membranes were blocked with 5% nonfat dried milk in PBS containing 0.1% Triton X-100 and then incubated with primary antibodies [anti-Tollip (abcam) and anti-NeuN, anti-DCX, anti-GAPDH (Santa Cruz Biotechnology)] at 4°C overnight. Specific secondary antibodies were used at a dilution of 1:5,000 and signals were visualized by LAS500 (GE Healthcare). Image J was used for relative intensity analysis. 

### Immunofluorescence staining

Immunofluorescence analyses were performed on mouse brains with DAPI, anti-Tollip (1:50; abcam), and anti-Ki-67 (1:50; Santa Cruz Biotechnology) antibodies. Mice were anesthetized with CO2 gas, and their brains were removed and fixed with paraformaldehyde solution at 4°C overnight. Sections were fixed and rinsed, followed by incubation in blocking solution (2.5% BSA in PBS), rinsing in PBS, and then incubation in primary antibody solution (containing 0.1% Triton-X 100 and 1% BSA) overnight at 4°C. 

### Statistics

All statistical analyses were performed using Prism 5.0. Unpaired student's t-tests were used for two-group comparisons. Statistical analysis was performed by using student's t-tests. One-way ANOVAs were used for western blotting intensity analysis and the Neuro2A cell viability test followed by Bonferroni's post hoc tests. P values of <0.05 were considered significant.

## RESULTS

### Analysis of SGZ-specific gene expression changes after voluntary running and comparison with data from an Autism mouse model

**Figure 1.**
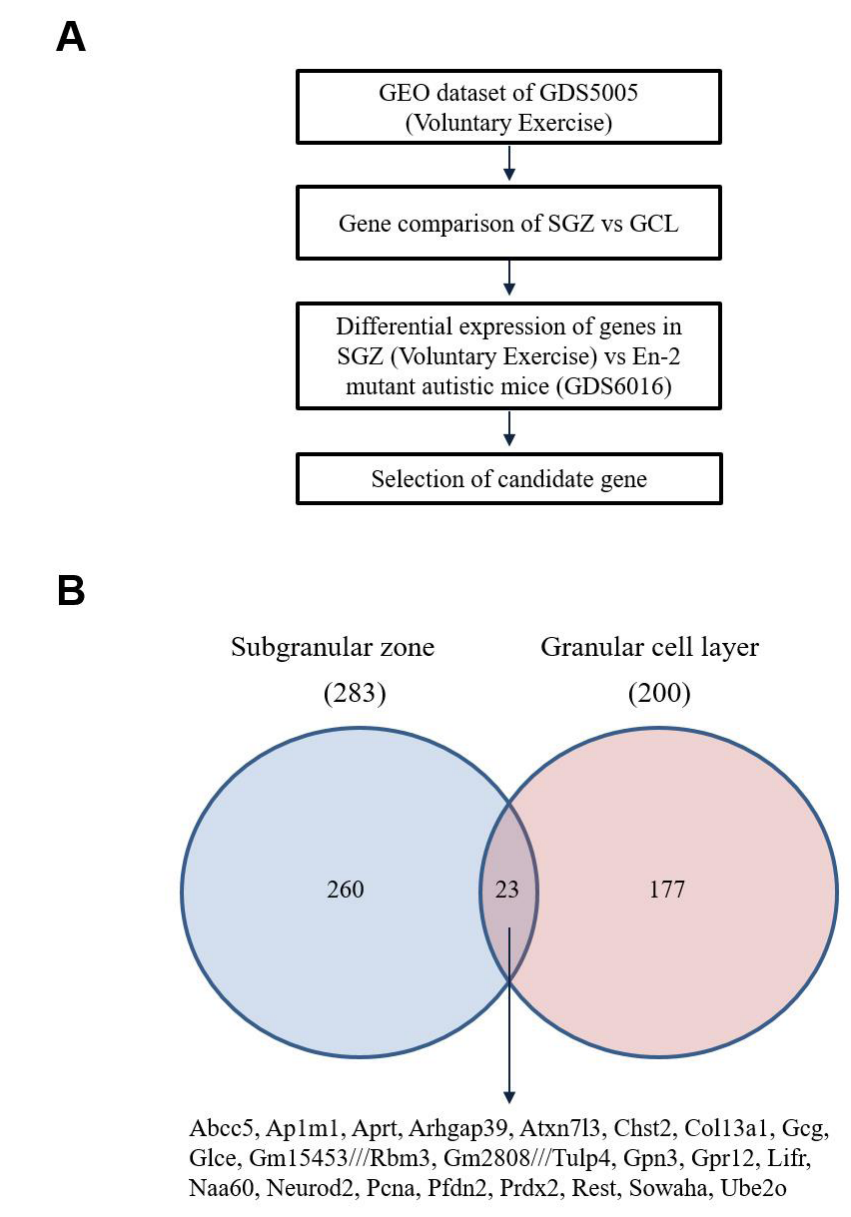
(A) Scheme for comparing gene expression in the SGZ vs the GCL in exercised mice and also the SGZ of exercised mice and the hippocampi of En2 mutant mice. Diagram shows the comparison of genes changed by exercise in the SGZ and GCL. (B) Diagram depicting genes that have changed expression levels following voluntary exercise in the SGZ and GCL, and their overlap.

To compare exercise-induced differential expression of genes in the GCL and SGZ of the DG, we first analyzed a microarray dataset of segmented mouse hippocampi after voluntary exercise (GDS5005, Table 1). There were 283 changes in gene expression in the SGZ, and 200 changes in the GCL of mouse hippocampi after voluntary exercise (30 days of wheel running), with an overlap of only 23 genes (Fig. 1). These commonly altered candidate genes were further compared with genes changed in the hippocampi of engrailed-2 (*En2*) mutant mice which show an autism-like phenotype (Table 1). Eighteen of the candidate genes were changed in both the SGZ of running mice and the hippocampi of En2 mutant mice, and five genes showed different patterns of expression in each condition. 

**Table 3.** Common genes changed in autistic disorders model (GDS6016) and voluntary exercise (GDS5005); red indicates up-regulated genes and dark blue indicates the down-regulation genes compared to each control group.

	Gene	En2_autism	30day Exercise (SGZ)	Description
1	Bcat2			branched chain aminotransferase 2
2	Bckdhb			branched chain ketoacid dehydrogenase E1,
3	Cacng2			calcium channel, voltage-dependent, gamma subunit 2
4	Ccl25			chemokine (C-C motif) ligand 25
5	Cdkn2d			cyclin-dependent kinase inhibitor 2D (p19, inhibits CDK4)
6	Cplx2			complexin 2
7	Dnajc5			DnaJ heat shock protein family (Hsp40) member C5
8	Gys1			glycogen synthase 1
9	Miip			migration and invasion inhibitory protein
10	Papss2			3'-phosphoadenosine 5'-phosphosulfate synthase 2
11	Pnkd			paroxysmal nonkinesiogenic dyskinesia
12	Rufy1			RUN and FYVE domain containing 1
13	Sf3b3			splicing factor 3b, subunit 3
14	Sptbn2			spectrin beta, non-erythrocytic 2
15	Tollip			toll interacting protein
16	Ube2m			ubiquitin-conjugating enzyme E2M
17	Zfr			zinc finger RNA binding protein

### Expression of *Tollip *after voluntary exercise

**Figure 2.**
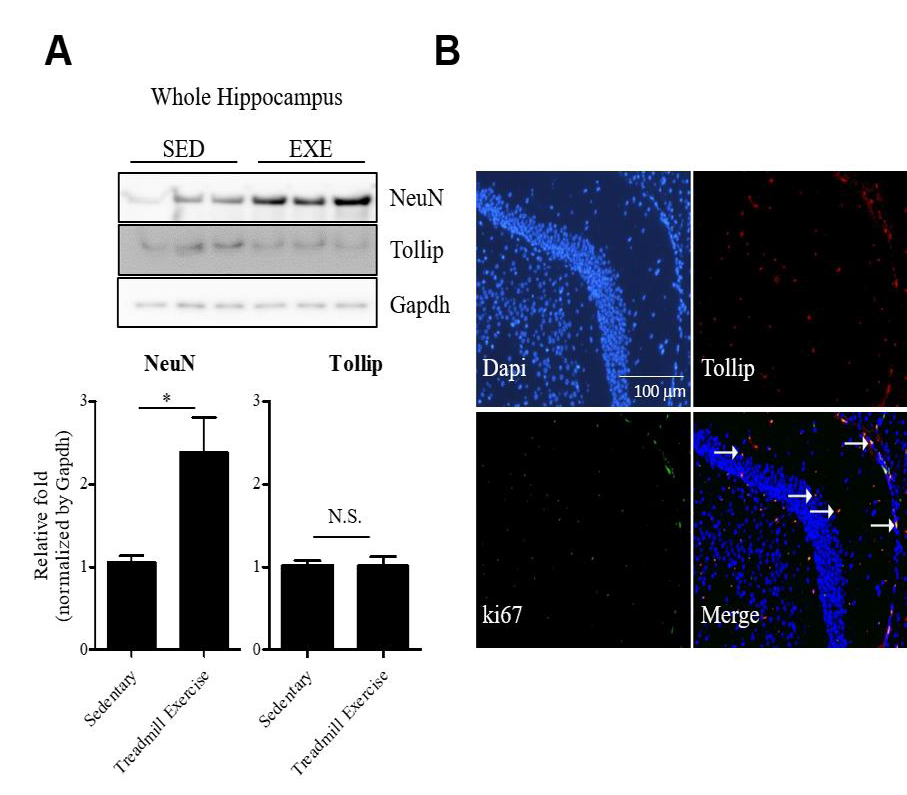
(A) Scheme for comparing gene expression in the SGZ vs the GCL in exercised mice and also the SGZ of exercised mice and the hippocampi of En2 mutant mice. Diagram shows the comparison of genes changed by exercise in the SGZ and GCL. (B) Diagram depicting genes that have changed expression levels following voluntary exercise in the SGZ and GCL, and their overlap.

Among the five genes, Tollip has been linked to neurodegeneration and neuroinflammation^31^, so we further examined the levels of Tollip by immunoblot analysis. The levels of Tollip protein were measured in the hippocampi of mice that ran on treadmills for two weeks. Running does not increase the amount of Tollip in the whole hippocampus sample compared to sedentary mice (Fig. 2A) (*p*>0.05). Then we investigated the presence of Tollip in the DG utilizing a Tollip-specific antibody and a Ki-67 antibody, a marker used for evaluating cell proliferation in the DG, as shown in Fig. 2B. The merged picture shows that there are Tollip and Ki-67 co-labelled cells in the DG area of the hippocampus Fig. 2B). 

### Effect of *Tollip*-deficiency on neurogenesis-related genes

**Figure 3.**
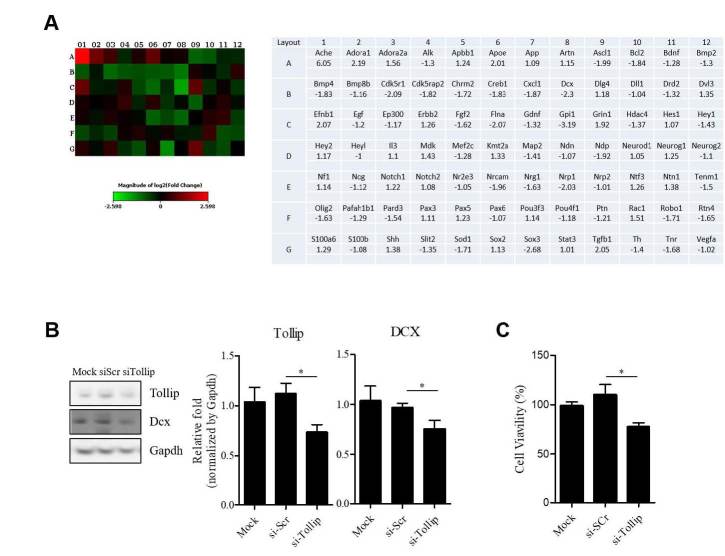
(A) Heat maps representing the relative expression levels of all genes involved in neurogenesis in Neuro2A cells. Comparison of cells transfected with Tollip siRNA compared to control siRNA. Below: The list of selected genes based on fold change from PCR array (compared to si-scramble transfected cells). (B) Expression levels of Tollip and DCX in Neuro2A cells after Tollip siRNA or scramble siRNA transfection. Quantitative immunoblotting analysis was normalized by GAPDH (n=5). DCX: 45 kDa, Tollip: 30 kDa, GAPDH: 37 kDa. (C) CCK-8 analysis of the effect of knockdown of Tollip on Neuro2A survival at 72 hours after transfection. NS, not significant. Error bars show mean ± SE. *P < 0.05 vs. scramble siRNA

We further investigated the expression patterns of genes involved in neurogenesis and in neuronal stem cells using PCR array (Qiagen) after transfecting Neuro2A cells with *Tollip* siRNA (Fig. 3A). When we applied a two-fold cut-off, five genes (*Ache*, *Adora1*, *ApoE*, *Efnb1*, and *Tgfb1*) were upregulated and five genes (*Cdk5r1*, *Dcx*, *Flna*, *Nrp1*, and *Sox3*) were downregulated in the *Tollip* knockdown cells compared to control cells transfected with scramble siRNA. Next, we validated and assessed the protein levels of DCX, a well-established marker for neuronal migration and axonogenesis by western blot analysis. A post-hoc test following one-way ANOVA analysis indicated that neuronal cells transfected with Tollip siRNA have decreased levels of DCX compared to control cells (p<0.05) (Fig. 3B). 

### Effect of *Tollip* knockdown on Neuro2A cell growth

The co-localization of Ki-67 and Tollip in the DG (Fig. 2B) drove us to study the effect of Tollip on neuronal cell viability. We used a cell counting kit-8 (CCK-8) assay to assess the viability of Neuro2A cells. *Tollip* siRNA knockdown cells have decreased cell viability compared to scramble siRNA transfected control cells (*p*<0.05) (Fig.3C).

## DISCUSSION

Analysis of microarray data from the hippocampal SGZ and GCL of exercised mice shows that there are quite a few differences within the hippocampus after 30 days of voluntary exercise. Differences in the expression levels of Tollip analyzed by western blot using whole hippocampi samples and the immunofluorescence data in the DG suggest that we should be cautious in interpreting data from the hippocampus, particularly when performing mechanistic studies. We also found that Tollip deficiency exerts a neurodegeneration-like phenotype by decreasing cell viability and DCX levels in Neuro2A cells.

Many studies have focused on the role of the hippocampus in mediating the effects of exercise on mood regulation and cognitive function^15-17,19^. Biochemical and omics studies suggest that exercise-modified factors such as BDNF, NPY, MIF, and NGF may be related to brain plasticity and neurogenesis. However, these data only partially covered the physiology of brain function, and thus their roles in mood regulation^32^, learning, and memory behaviors^33^ are controversial. Recent studies have revealed that different areas within the hippocampus may have different genetic characteristics^22,23^. We analyzed previous microarray data and compared gene expression in the hippocampal SGZ and GCL after exercise. We found only approximately 10% of genes were commonly affected by exercise between the two different areas of the hippocampus. These results indicate that previous studies regarding exercise-mediated molecular changes in the hippocampus may need to be reconsidered depending on the location within the hippocampus. 

Due to the observed similarities between autism improvements and the effects of exercise^9,11^, we looked for genes changed by exercise in the SGZ and genes that are reversed in En2 knockout mice^30^. Tollip is an inhibitory adaptor protein within Toll-like receptors (TLR), and a recent study suggested a link between *Tollip* deficiency and neurodegeneration in mice^31^. We found that Tollip was increased only in the SGZ, but not in the GCL. We also found that levels of Tollip were not increased in the whole hippocampus, but co-localized with Ki-67, a proliferation marker in the DG. One of the causes of this result is probably the type of exercise studied. Although both voluntary exercise and involuntary movement show neurogenic effects (18, 20), the precise timing of the effect on the brain and the biomechanical mechanisms may differ^34^. To clarify these results, we performed cell viability tests and found that the number of neuronal cells decreased with *Tollip* deficiency. PCR-array data indicate that DCX may be affected by the amount of Tollip. Indeed, *Tollip*-knockdown decreased the levels of DCX protein, an immature neuronal marker, in Neuro2A cells. 

These data suggest that Tollip may mediate the beneficial effects of exercise on neuronal cell survival and functions in the brain, specifically in the SGZ. Finally, these results indicate that it is necessary to consider regional differences when studying the effects of exercise on the brain.
